# Correlation of cyclin D1 expression with aggressive DNA pattern in patients with tobacco-related intraoral squamous cell carcinoma

**Published:** 2011-04

**Authors:** Satya N. Das, Pratima Khare, Manoj K Singh, Suresh C. Sharma

**Affiliations:** Department of Biotechnology, All India Institute of Medical Sciences, New Delhi, India; * *Department of Pathology, All India Institute of Medical Sciences, New Delhi, India*; ** *Department of Otorhinolaryngology, All India Institute of Medical Sciences, New Delhi, India*

**Keywords:** Cyclin D1, DNA ploidy, oral cancer

## Abstract

**Background & objectives:**

Cyclin D1 has been strongly implicated in cell proliferation particularly in the G1/S checkpoint of the cell cycle, and prognoses in human malignancies. We investigated the correlation between cyclin D1 overexpression and clinicopathological features as well as cell cycle parameters to understand its clinical significance in patients with tobacco-related oral squamous cell carcinoma (OSCC).

**Methods:**

Immunohistochemistry for cyclin D1 and DNA flowcytometry for cell cycle parameters was done on paraffin embedded tumour samples from 45 patients with OSCC

**Results:**

Higher expression of cyclin D1 was observed only in 30 (66.6%) of 45 cases that correlated with advanced age (*P* <0.02), higher tumour stage ((*P*<0.01), histological differentiation and lymph node metastasis (*P* <0.01). Analysis of nuclear DNA pattern revealed cyclin D1 immunoreactivity in tumours with aggressive DNA pattern such as aneuploidy ((*P*<0.05) and higher S phase fraction ((*P*<0.04).

**Interpretation & conclusions:**

Higher expression of cyclin D1 in oral cancer appears to be closely linked to cell proliferation, differentiation and lymph node invasion. Pre-operative evaluation of cyclin D1 in biopsy specimen may be useful in planning the most appropriate treatment strategies in patients with tobacco-related OSCC.

Tobacco-related oral squamous cell carcinoma (OSCC) is one of the most common malignancy affecting males in India. Of the 400,000 annually reported new cases world over, 100,000 oral squamous cell carcinoma cases occur in India^1^ alone. Cancer of the oral cavity presents certain special problems. These tumours are slow growing, remain localized for longer time, and then spread to regional lymph nodes. Distant metastases are uncommon. However, most patients present with advanced stage lesions with multiple lymph nodes that are beyond reach of surgery and radiotherapy^2^. In addition, variability in the clinical course of the disease possibly due to poor monitoring of OSCC in absence of a reliable biomarker, increases morbidity and mortality. Therefore, identification and defining suitable biomarker that could provide prognostic assessment of the disease, would help in designing most appropriate treatment strategies for OSCC and monitoring the disease activity during follow up period.

A frequent target in carcinogenesis is the deregulation of G1 to S phase progression of the cell cycle^3^. The transition through G1 to S phase is regulated by cyclins, cyclin-dependent kinases (CDK)-CDK4 and CDK6 and their inhibitors. Cyclin D1 is a key regulatory protein at G1/S checkpoint of the cell cycle. It forms complexes with CDK4 or CDK6 and is responsible for the phosphorylation of the retinoblastoma tumour suppressor protein, resulting in the release of E2F transcription factors that allow cell to enter into S phase^4^. The G1/S checkpoint is frequently altered in many epithelial tumours and may confer growth advantage and enhanced tumorigenesis. Consistent with its positive effect on cell cycle progression, cyclin D1 (prod-1, bcl-1) qualifies as a proto-oncogene^5^. Amplification and overexpression of cyclin D1 has been reported to be more frequent in head and neck^6^, oral^7^–^9^, laryngeal^10^ and nasopharyngeal carcinoma1^11^,^12^. Earlier we reported higher frequency of aneuploidy and higher S-phase fraction in oral tumours that was related to advanced clinical stage, lymph node metastasis, poor histological differentiation and early recurrence^13^. However, reports on cyclin D1 expression and their relation to clinicopathological features and cell cycle parameters in tobacco-related oral cancer are scanty. Therefore, the present study was planned to study cyclin D1 expression in patients with tobacco-related intraoral squamous cell carcinoma and their correlation with clinicopathological features and aggressive DNA pattern as determined by DNA flow cytometry.

## Material & Methods

*Patients:* This retrospective study was done on tumour samples collected from 45 patients with intraoral squamous cell carcinoma operated at the Department of Otorhinolaryngology, All India Institute of Medical Sciences (AIIMS), New Delhi between 2001-2006. The study protocol was approved by the ‘Ethics Committee’ of the AIIMS and written consent was obtained from all the study subjects. Age of the patients ranged from 27 to 75 yr with a mean age of 53.2 ± 12.2 yr; 36 (80%) patients were males while 9 (30%) were females. All the patients had history of tobacco chewing for periods ranging from 5 to 25 yr. The most commonly affected sites were lower alveolus, buccal mucosa and tongue, followed by other sites like lower lip, retromolar trigone G.B. sulcus and floor of the mouth. None of the patients had received pre-operative radiation or chemotherapy before the biopsy was taken. Tumour, node and metastasis (TNM) classification and clinical staging of the tumour were done as per criteria laid down by American Joint Committee on Cancer^14^. Since very few patients had T1 and T3 tumours, all were divided into two groups T1/T2 and T3/T4 for the analysis of data.

*Histopathological examinations:* Histopathological analysis was performed on primary tumours on haematoxylin and eosin stained sections. Histological grade was determined as per standard criteria^14^. As per this criteria, 31 (68.8%) patients had well differentiated tumour, 14 (31.1%) presented with moderately differentiated and none with poorly differentiated tumour. Paraffin blocks containing more than 70 per cent of tumour area were selected for sectioning for immunohistochemical and flowcytometric studies.

*DNA flowcytometry:* Flowcytometry was performed on nuclei prepared from 30 μm thick sections from formalin fixed paraffin embedded tissue as described earlier^13^, by the modified technique of Hood *et al*^15^. Briefly, sections were deparaffinized with xylene and re-hydrated with changes in graded ethanol followed by two washes with phosphate buffered saline (PBS, 0.1 M, *p*H 7.4). The samples were subjected to chemical digestion by incubating with 0.5 per cent pepsin (*p*H 1.5; Sigma, USA) at 37° C in water bath for 30 min with intermittent stirring. Disaggregated tissues were filtered through a 50 μM nylon mesh, washed twice with PBS and re-suspended in PBS. The cell suspension was fixed by adding cold ethanol and labelled with 50 μg/ml propidium iodide (PI) with 0.01 per cent Triton X and RNAse (1.0 mg/ml, Sigma, USA) at room temperature for 45 min in the dark. The samples were acquired in flowcytometer (FacScan, Becton- Dickinson Inc., San Jose, CA) equipped with 488 nm 15 mW argon ion laser. Calibration of flow cytometer was done using PI labelled chicken RBC (Becton-Dickinson, USA). Normal human peripheral blood lymphocytes were used to identify the normal diploid peak that served as reference peak for subsequent assays.

At least 10,000 events were collected for each sample using ‘Cell Quest’ software (Becton-Dickinson Inc., San Jose, CA). Acquisition program was set to collect DNA fluorescence signal as total area versus peak (signal height) in order to eliminate doublets and aggregates. Data were analyzed using ‘Modfit LT’ software (Becton Dickinson Inc., San Jose, CA). Mean co- efficient of variation of the measurements were within acceptable range (2-8%) in all the samples.

*Immunohistochemistry for cyclin D1:* The cyclin D1 expression was studied in formalin-fixed and paraffin embedded tumour samples by the standard immunohistochemical technique on 5 micron paraffin sections using Streptavidin – biotin Universal Detection system (Immunotech, France). Briefly, after sequential re-hydration through acetone, ethanol and distilled water, the endogenous peroxidase activity was blocked using 3 per cent H_2_O_2_ in methanol at room temperature for 5 min. The sections were washed with water and antigen was retrieved by heating sections in microwave (700W) in 10 mM citrate buffer (*p*H 6.0) for 20 min. Before incubation in antibody solution, the sections were covered with protein blocking agent (PBA) for 5 min at room temperature, excess PBA was taped off and sections were covered with mouse monoclonal anti-cyclin D1 antibody (1:100 dilution; Clone-5D4, Immunotech, France) for 2 h in a wet chamber. The sections were washed and treated with biotinylated secondary antibody (anti-mouse 1gG) for 30 min at room temperature followed by treatment with streptavidin-peroxidase for 30 min at room temperature. The colour was developed using freshly prepared chromogen (DAB). The sections were counter stained with Mayer’s haematoxylin and mounted with DPX mountant. Negative control slide was prepared similarly by omitting primary antibody. Slides were examined under light microscope and scored positive in case with brown nuclear staining in >5 per cent cells in three randomly selected areas.

*Statistical analysis:* Statistical analysis was performed by STATA-7.0 (intercooled version) computer software (Stata Inc. Houston, TX, USA) using two-tailed Fisher’s exact test. Statistical significance was defined as (*P*<0.05.

## Results

**Fig. 1:**
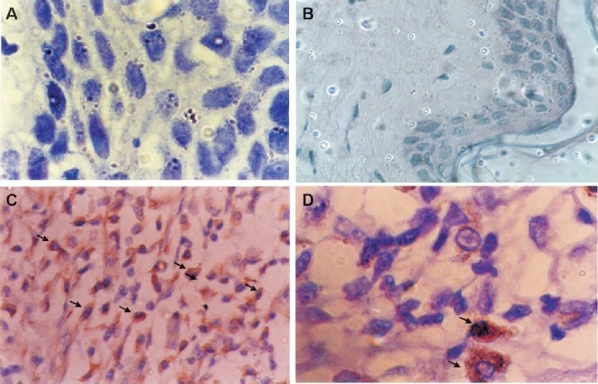
Immunohistochemical staining of cyclin D1 in tobacco-related intraoral squamous cell carcinoma using mouse monoclonal antibody; **(A)** negative control and **(B)** normal buccal mucosa are negative for cyclin D1; **(C)** moderately differentiated tumour cells show strong cytoplasmic and nuclear (arrows) immunoreactivity (magnification (40X); **(D)** shows intense nuclear and cytoplasmic immunoreactivity in tumour cells (arrow) under higher (100X) magnification.

Of the 45 patients, 30 (66.6 %) showed cyclin D1 immunoreactivity while immunoreactivity was not seen either in negative control (Fig. 1A) or in normal tissues (Fig. 1B) suggesting its absence in normal tissues. Among patients, high cyclin D1 expression (>50% +ve nuclei) was observed in 20 (44.4%) cases (Fig. 1 C D), moderate expression (<;50% +ve nuclei) in 10 (22.2%) cases and the remaining 15 (33.3%) cases showed either low (<;5%) or no expression of cyclin D1. Thus, overall 66.6 per cent cases showed overexpression (high to moderate) of cyclin D1.

When cyclin D1 expression was analysed in relation to clinicopathological features of oral cancer patients (Table) it was found that expression cyclin D1 was significantly associated with advanced age (>50 yr) at the time of presentation ((*P*<0.02) higher tumour stage (T3, T4; (*P*<0.01) and lymph node metastasis ((*P*<0.01). Similarly, the frequency of cyclin D1 immunoreactivity was higher (78.5%) in patients with moderately differentiated tumours as compared to that with the well differentiated tumours (61.2%) although the difference between the two groups was statistically not significant.

**Table 1:** Cyclin D1 expression in tobacco-related oral carcinoma in relation to clinicopathological and cell cycle parameters

Parameters	No. of cases (n)	Cyclin D1 positive no. (%)
Age (yr)		
≤50	18	8 (44.4)
>50	27	22 (81.4)**
Gender		
Male	36	26 (72.2)
Female	9	4 (44.4)
Tumour stage		
T1, T2	13	5 (38.4)
T3, T4	32	25 (78.1)+
Lymph node metastasis		
Negative	23	11 (47.8)
Positive	22	19 (86.3)+
Histological differentiation		
Well	31	19 (61.2)
Moderate	14	11 (78.5)
Ploidy		
Diploid	20	10 (50.0)
Aneuploid	25	20 (80.0)*
S-phase fraction		
Low (<13%)	22	10 (45.4)
High (≥;13%)	23	20 (87.0)*

**Fig. 2:**
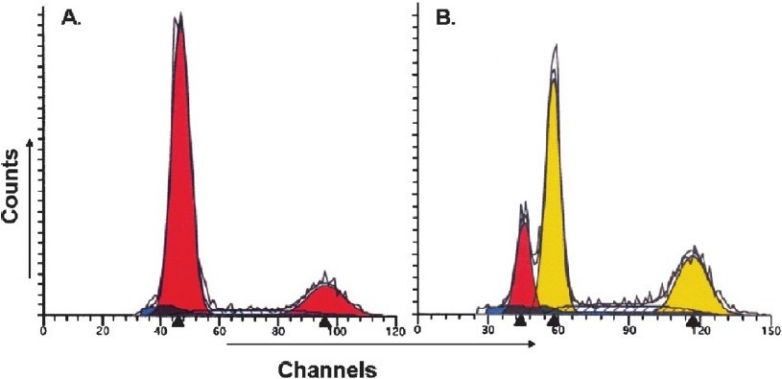
DNA histogram of tobacco-related intraoral squamous cell carcinoma analyzed by ‘Modfit LT’ software, showing **(A)** diploid tumour **(B)** aneuploid tumour.

* *P* < 0.05;

** < 0.02;

+ < 0.01;

The assessment of tumours by flowcytometry showed diploid tumours (Fig. 2A) in 20 patients (44.4%) and aneuploid tumours (Fig. 2B) in the remaining 25 patients (55.5%) while the mean value of S-phase fraction was 13.2 per cent. When analyzed in relation to cell cycle parameters, significantly higher ((*P*<0.05) frequency of patients (80.0%) with aneuploid tumour showed cyclin D1 immunoreactivity (Table) as compared to those with diploid tumours (50%). Similarly, significantly increased expression of cyclin D1 (87.0%; (*P*<0.05) was observed in patients with high SPF (>13%) as compared to those with low SPF (<;13.0%). Our results suggest a direct correction between cyclin D1 expression and aggressive DNA pattern (aneuploidy and high SPF) in patients with tobacco-related intraoral squamous cell carcinoma.

## Discussion

One of the advantages of immunohistochemical technique is that the overexpression of cyclin D1 can be detectable at single cell level and more importantly distribution of the overexpressing cells can be recognized among heterogeneous cellular population. In agreement with the previous studies^6^ reporting that the immunocytochemical technique detects only cells overexpressing cyclin D1 and does not detect cyclin D1 in normal tissues, present study demonstrated overexpression of cyclin D1 in 30 (66.6%) patients with tobacco-related OSCC while no expression was observed in the normal tissue. In majority of cases the immunoreactivity was localized to the nucleus of the tumour cells. Similarly, earlier studies have shown overexpression of cyclin D1 in 16-64 per cent of patients with head and neck squamous cell carcinoma^10^,^16^–^18^. Cyclin D1 has been characterized as an oncogene encoded by the *CCN D1* gene on chromosome 11q13^19^. Abnormal expression of cyclin D1 and CDKs has been considered to be one of the most important factors in the tumorigenesis of various human malignancies^20^. Amplification of the cyclin D1 gene was shown to be occurring in early stage of head and neck cancer and significantly associated with high proliferative activity^21^.

Studies of factors influencing patient’s outcome in head and neck tumours are problematic because of the heterogeneity of tumour stages at the time of diagnosis, tumour differentiation, site of tumour and lymph node involvement. One of the important points of the present study is that cyclin D1 expression in tobacco-related OSCC showed a significant correlation with clinicopathological features of patients and tumours as well. We found significantly higher frequency of overexpression of cyclin D1 in patients with advanced age, advanced tumours stage and lymph node metastasis. Furthermore, relatively higher frequency of cyclin D1 immuno-reactivity was also seen in patients with less differentiated tumours suggesting inverse correlation of cyclin D1 expression with histological differentiation of tumour. Similarly, increasing cyclin D1 immunoreactivity was observed through well to moderate and poorly differentiated tumours of patients with tobacco-mediated oral carcinoma^22^. The relationship between cyclin D1 and biologic behaviour of OSCC is not known. However, our results are in agreement with earlier studies that showed association of cyclin D1 overexpression with lymph node metastasis and advanced clinical stage in case with laryngeal squamous cell carcinoma^10^,^23^. Overexpression of cyclin D1 correlated with lymph node metastasis, poor histological differentiation and higher mitotic activity in patients with oesophageal carcinoma^24^.

Although details of the underlying mechanisms of cyclin D1 overexpression in the current study remain unclear, however, it appears to provide an advantage of tumour cells allowing higher proliferation and may promote tumour cells toward more advanced stages and cause metastasis to deeper tissues and regional lymph nodes. Thus strategies to prevent overexpression of cyclin D1 may be important for control of growth and metastasis of oral carcinoma. Recent study on nasopharyngeal carcinoma^25^ showed that overproduction of cyclin D1 is dependent on activation of mTOR (mammalian target for rapamycin) signaling pathway. Similarly, activation of STAT5-cyclin D1 pathway of signaling was reported in tobacco-related oral squamous cell carcinoma^26^. It has also been reported that overexpression of cyclin D1 correlates with sensitivity to cisplatin in squamous cell carcinoma cell lines of the head and neck, and antisense cyclin D1 enhances sensitivity to head and neck cancer cells to cisplatin^27^,^28^.

Another important finding of the present study was that aneuploid tumours and those with higher S phase fraction showed significantly higher frequency of cyclin D1 immunoreactivity as compared to diploid tumours and with lower S phase fraction. It seems, therefore, that overexpression of cyclin D1 is associated with aggressive DNA pattern such as aneuploidy and increased S phase fraction in tobacco-related OSCC. Our results confirm the hypothesis proposed by Staibano *et al*^7^, that analysis of cyclin D1 expression and DNA ploidy may help identifying basal cell carcinoma of head and neck with aggressive phenotype and poor outcome. We have earlier shown that aneuploidy and higher S phase fraction in metastatic tumour cells of oral carcinoma is associated with poor prognosis and low disease-free surviva1^13^. More interestingly, 3 to 4 times higher amplification of cyclin D1 has been reported in aneuploid breast cancer^29^. Aneuploidy and overexpression of cyclin D1 has been reported to be associated with shorter disease-free survival in basal carcinoma of head and neck^6^. These findings indicate that overexpression of cyclin D1 may be related to higher cellular proliferation and poor prognosis, thus it may serve as a potential tumour marker in patients with tobacco-related oral squamous cell carcinoma.

In conclusion, our results provide the evidence that cyclin D1 is overexpressed in a majority of patients with tobacco-related OSCC and that it is associated with aggressive clinicopathological features such as advanced age, tumour stage, lymph node metastasis as well as aggressive DNA pattern (aneuploidy and high S phase) in these patients. Therefore, evaluation of cyclin D1 expression in pre-operative biopsy specimen may be useful in prognosticating and planning most appropriate treatment strategies in patients with tobacco-related oral squamous cell carcinoma.
